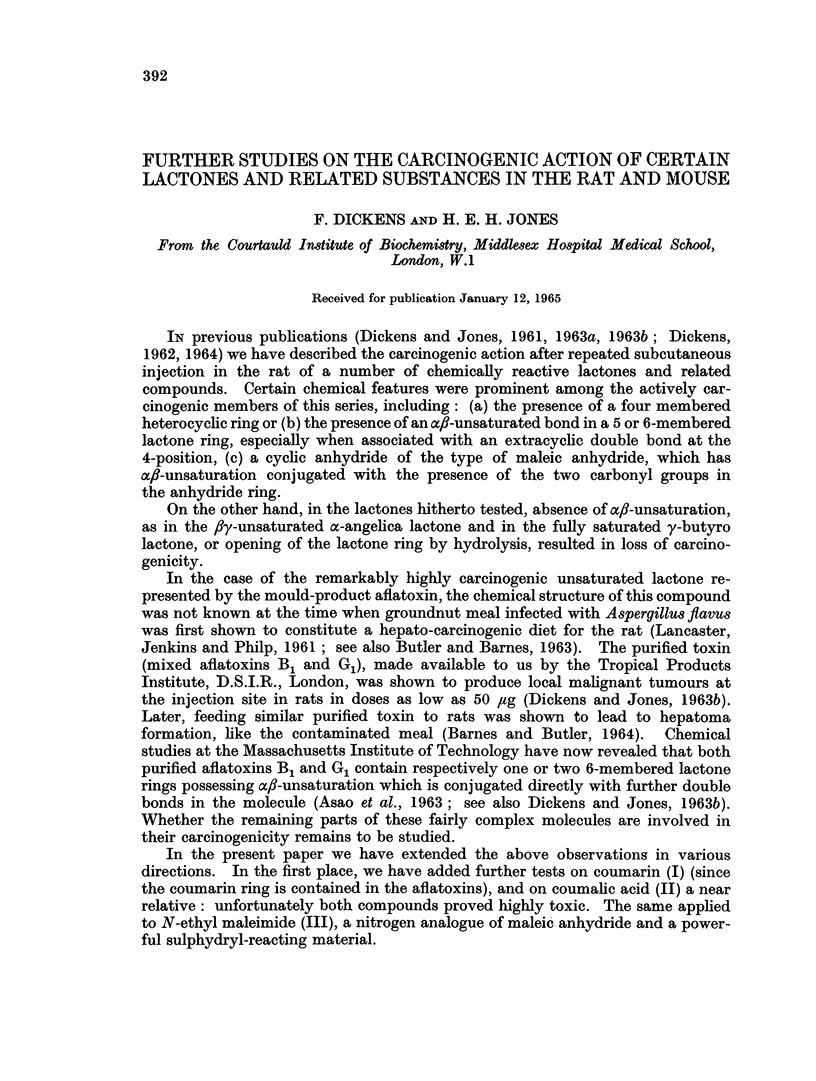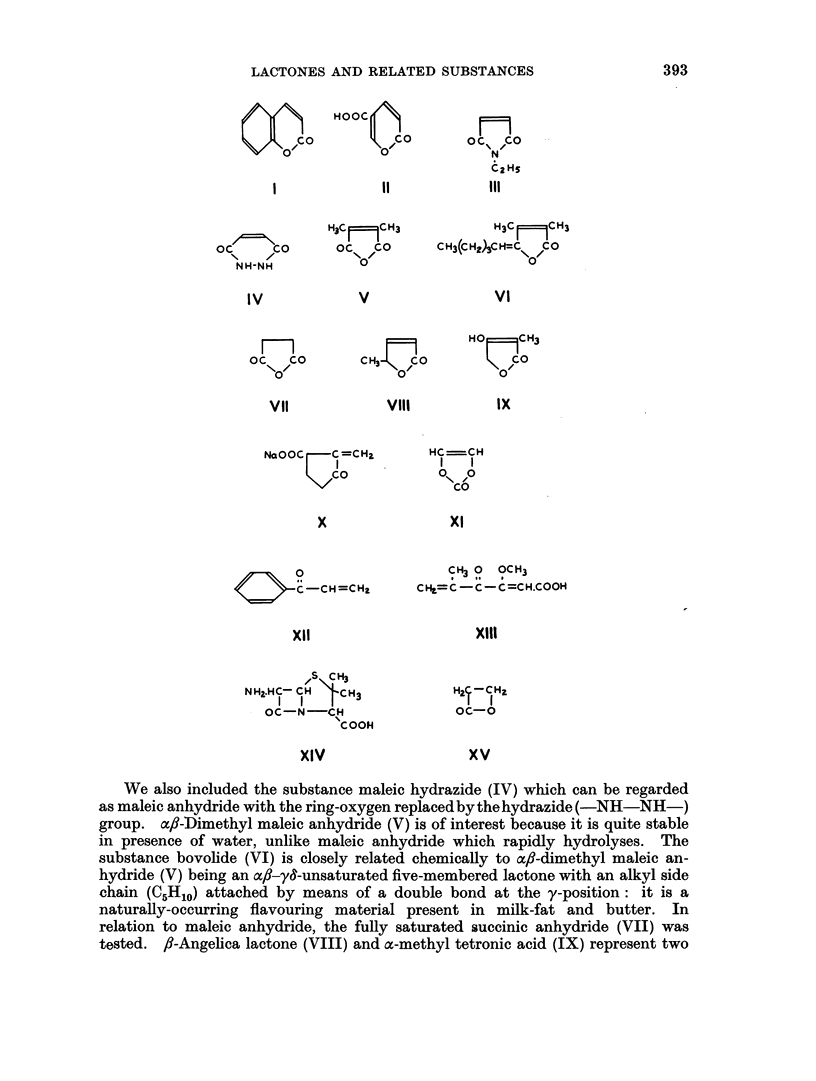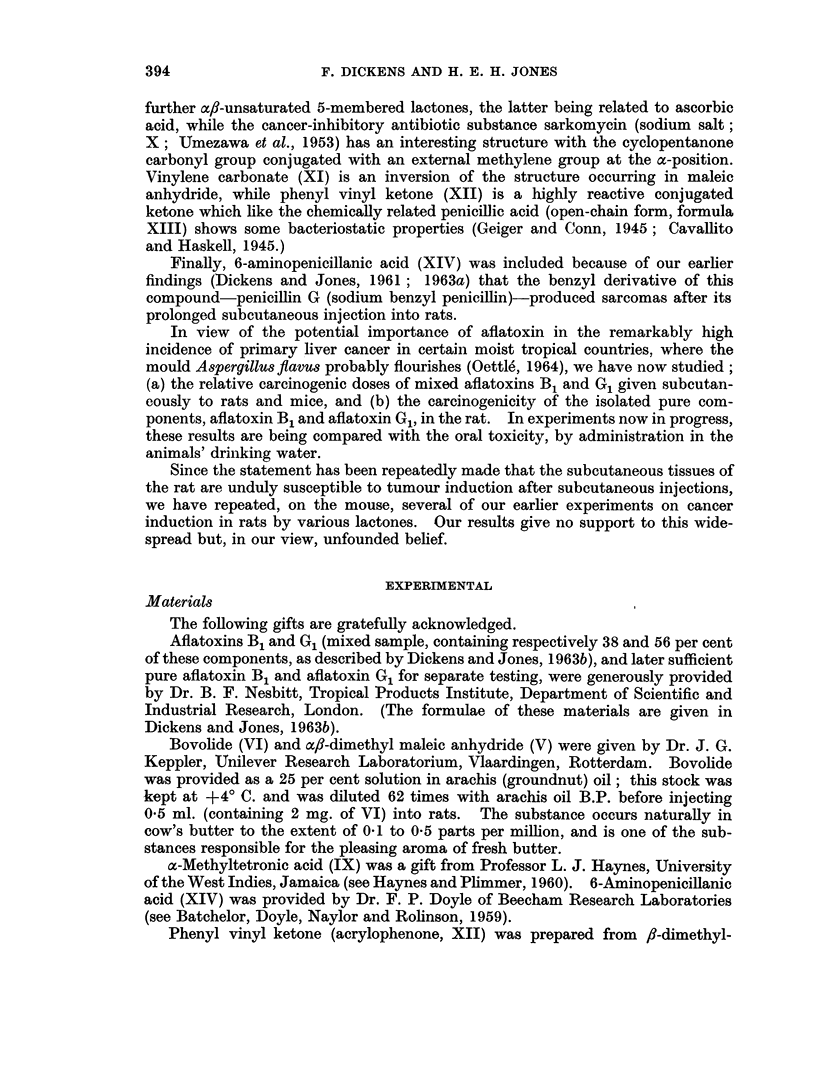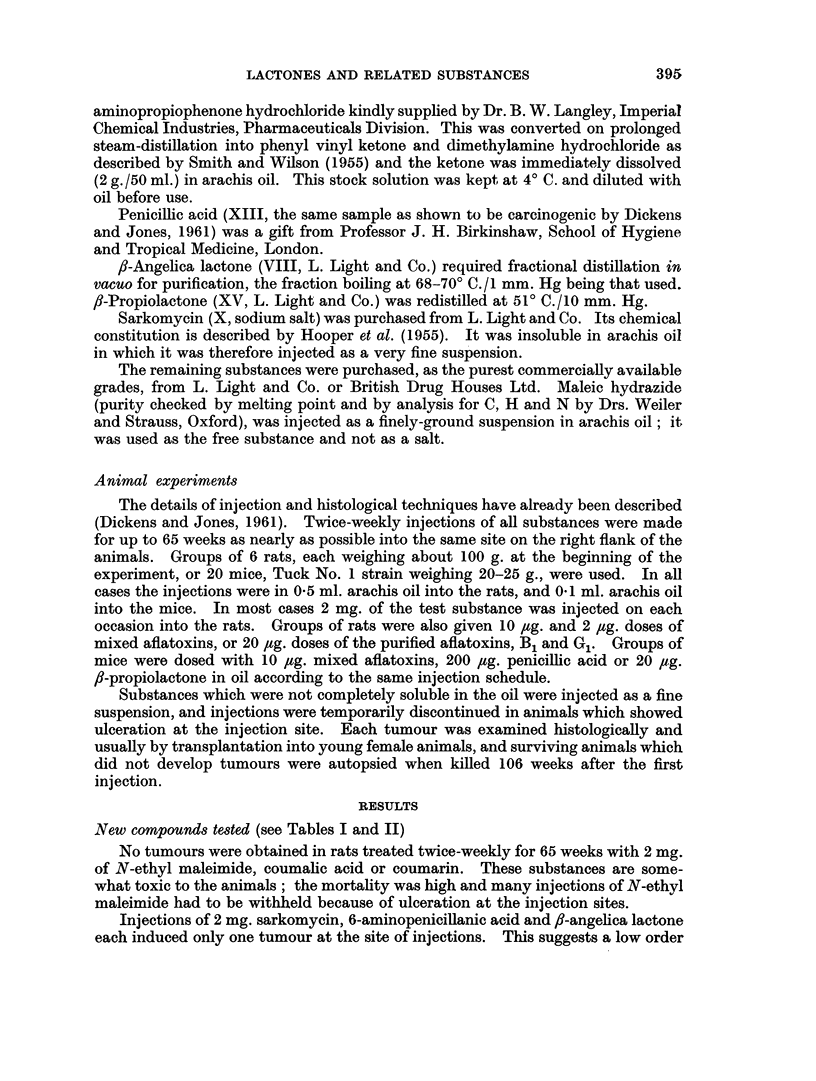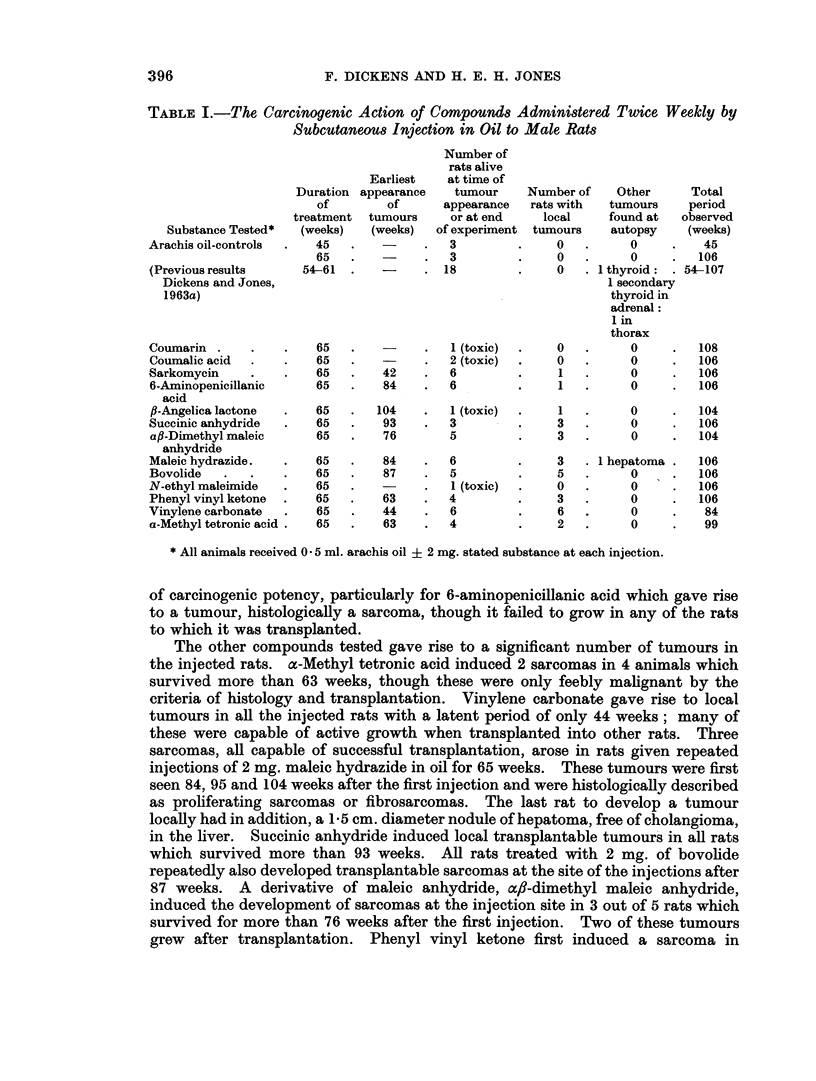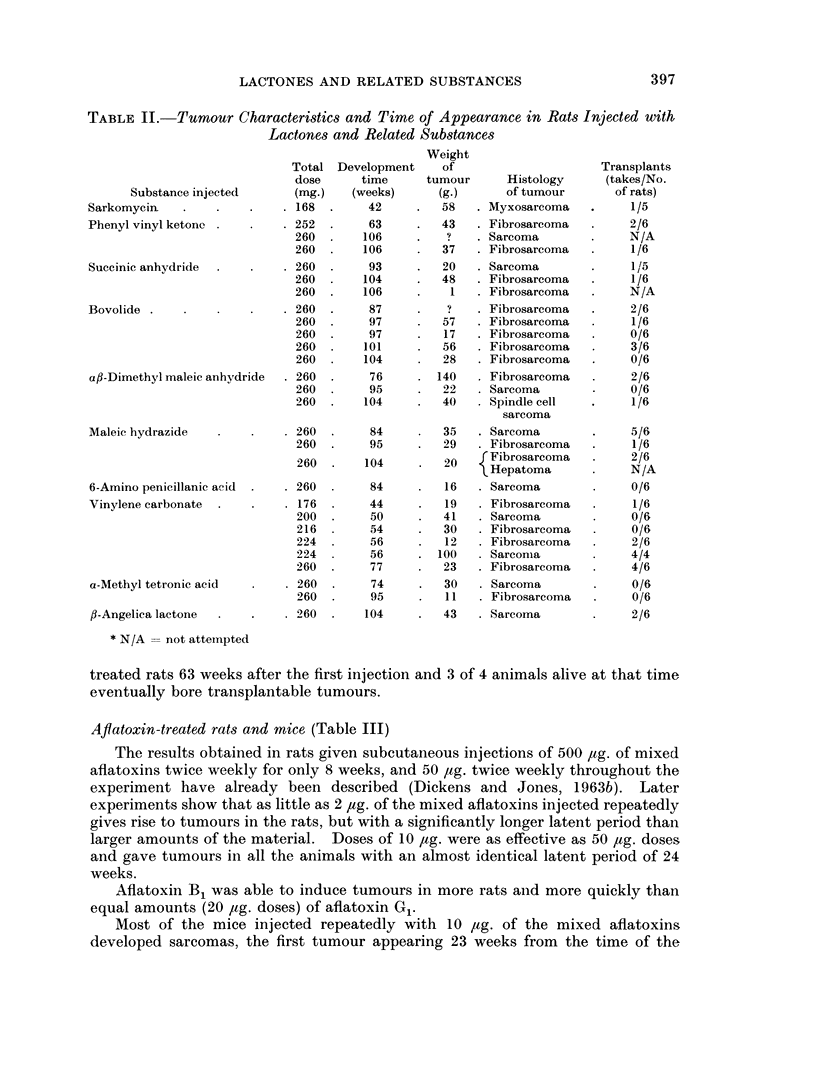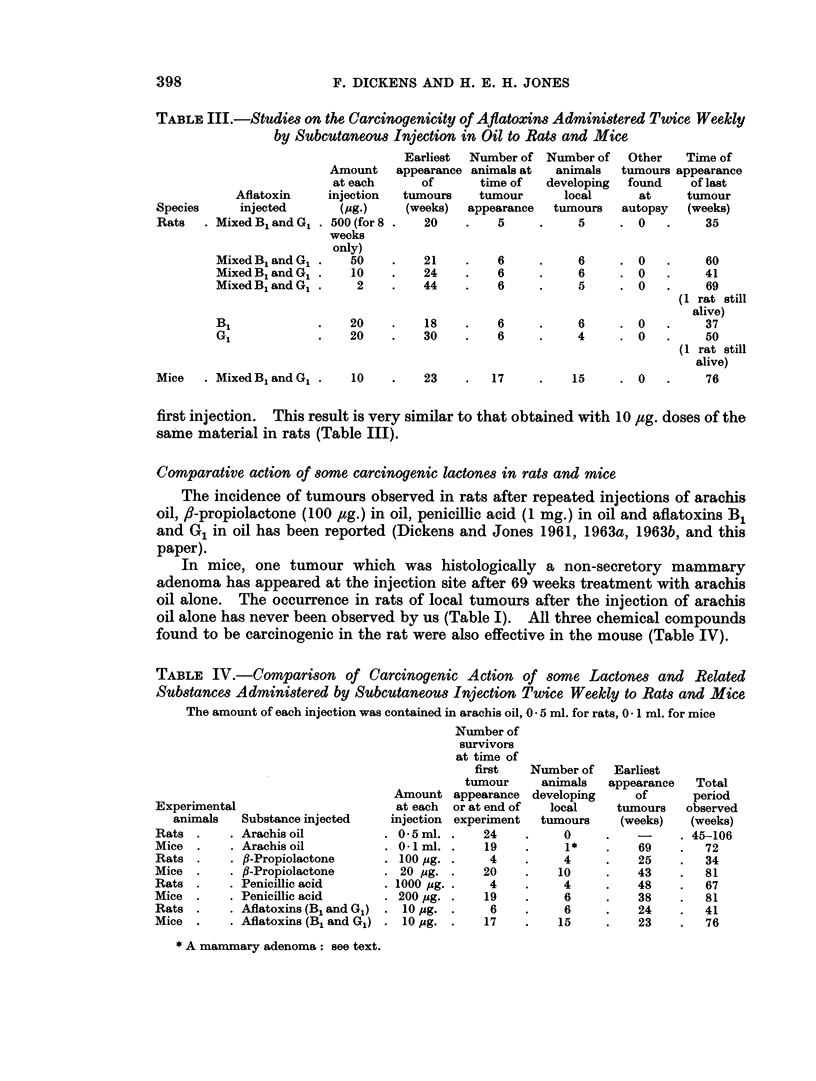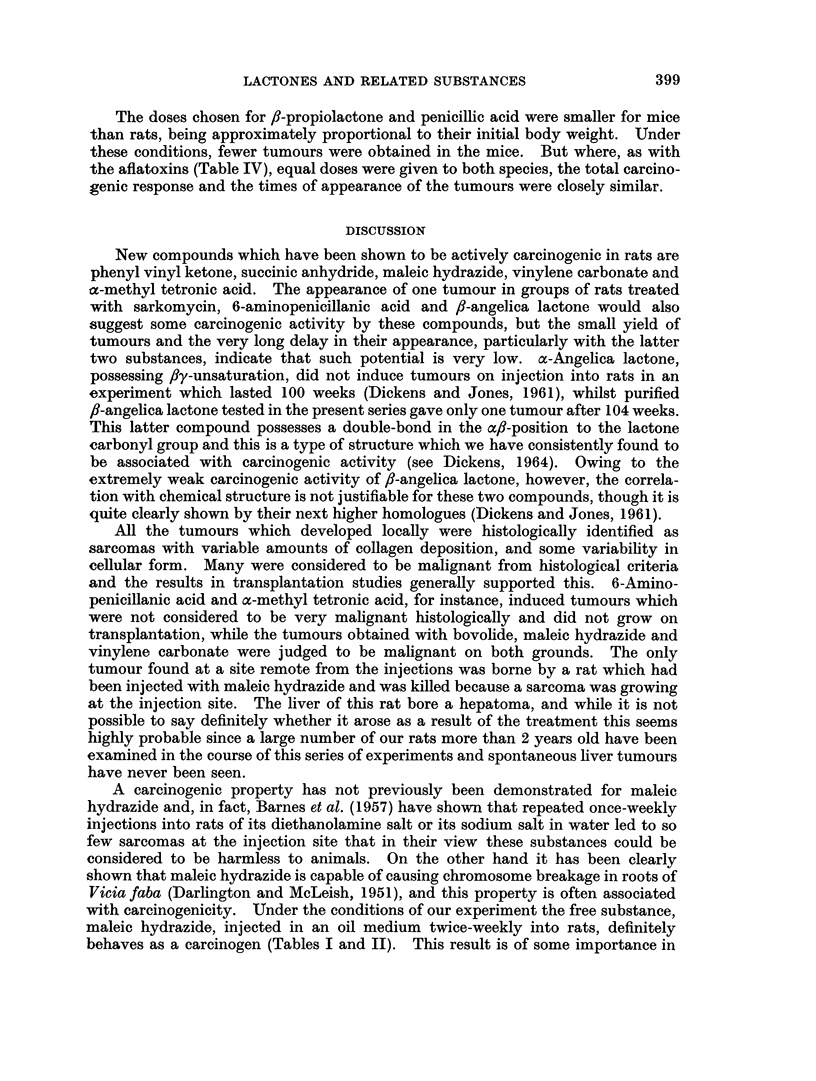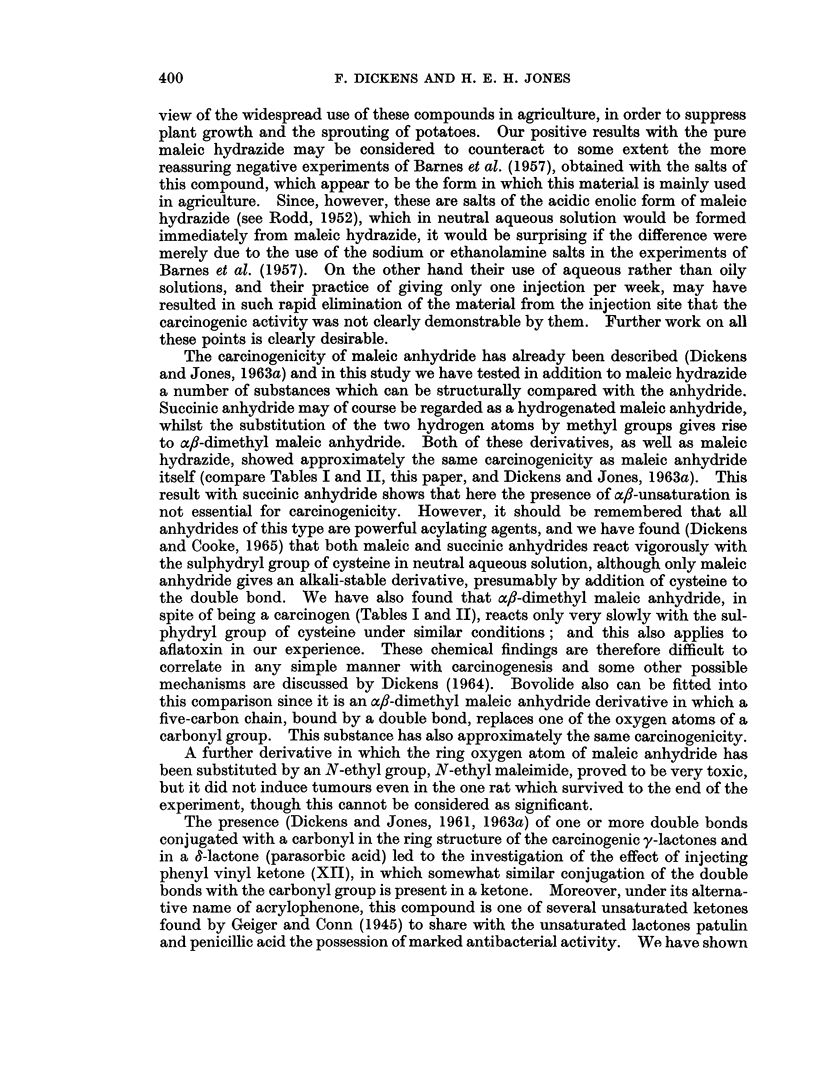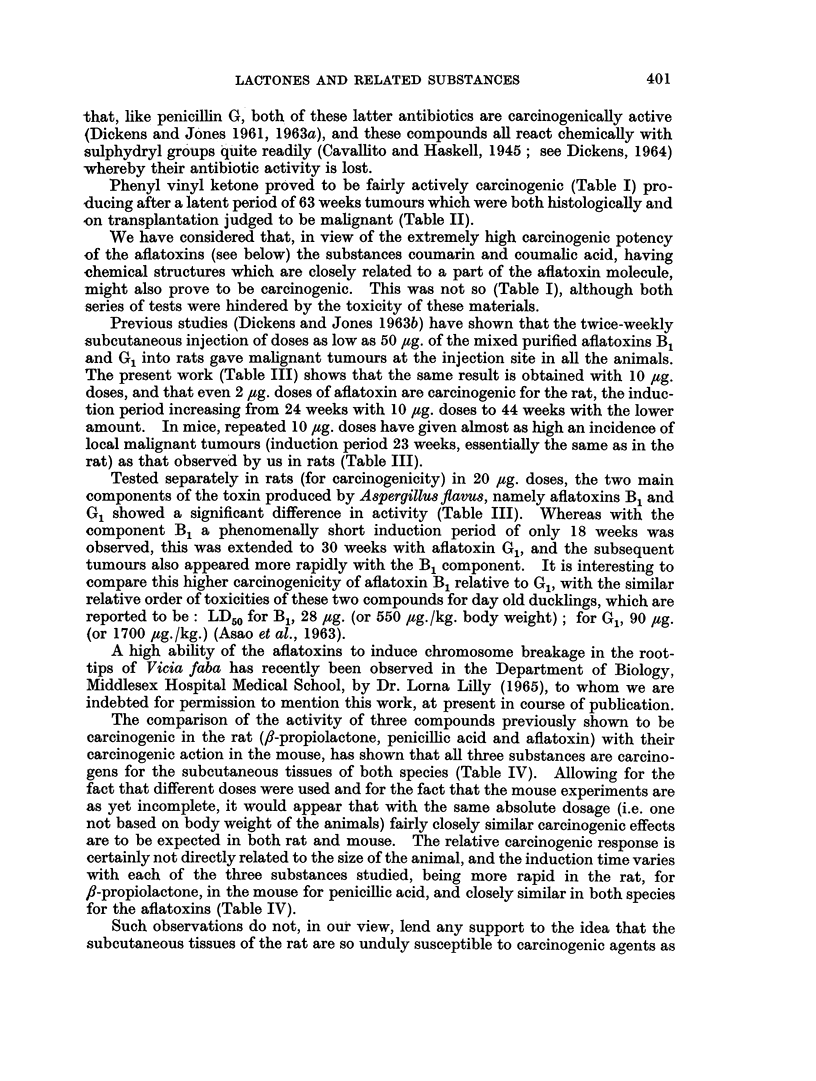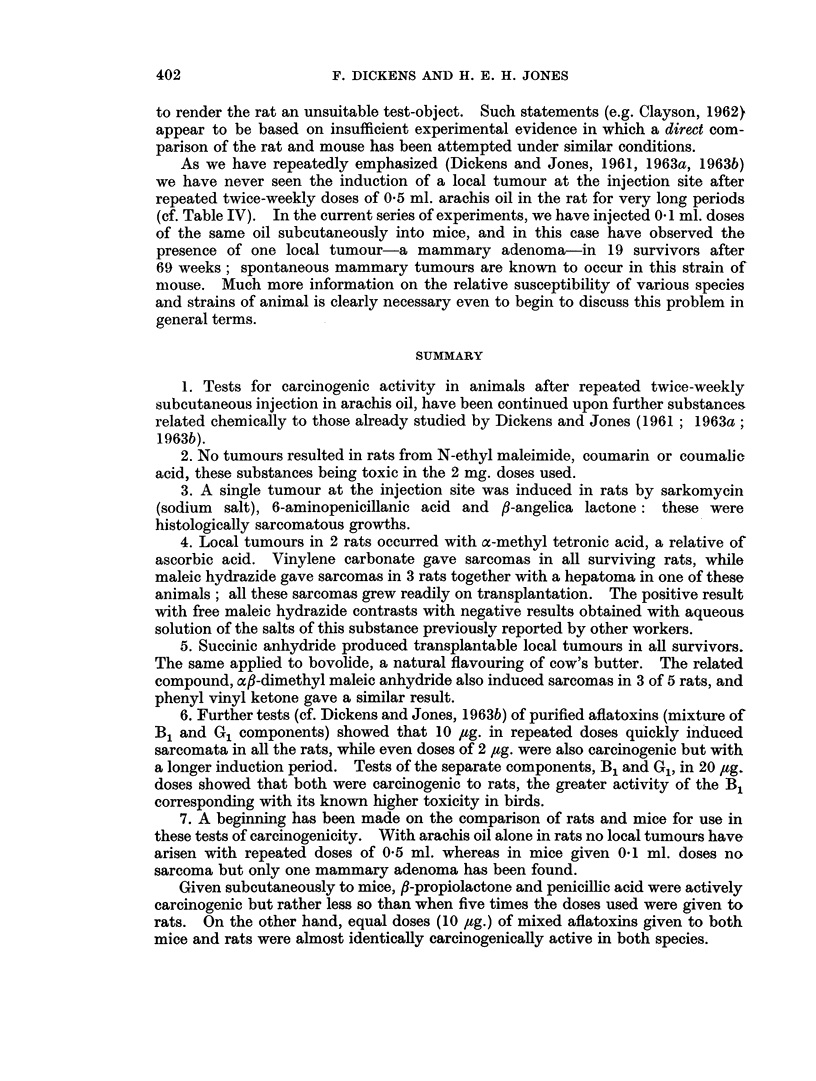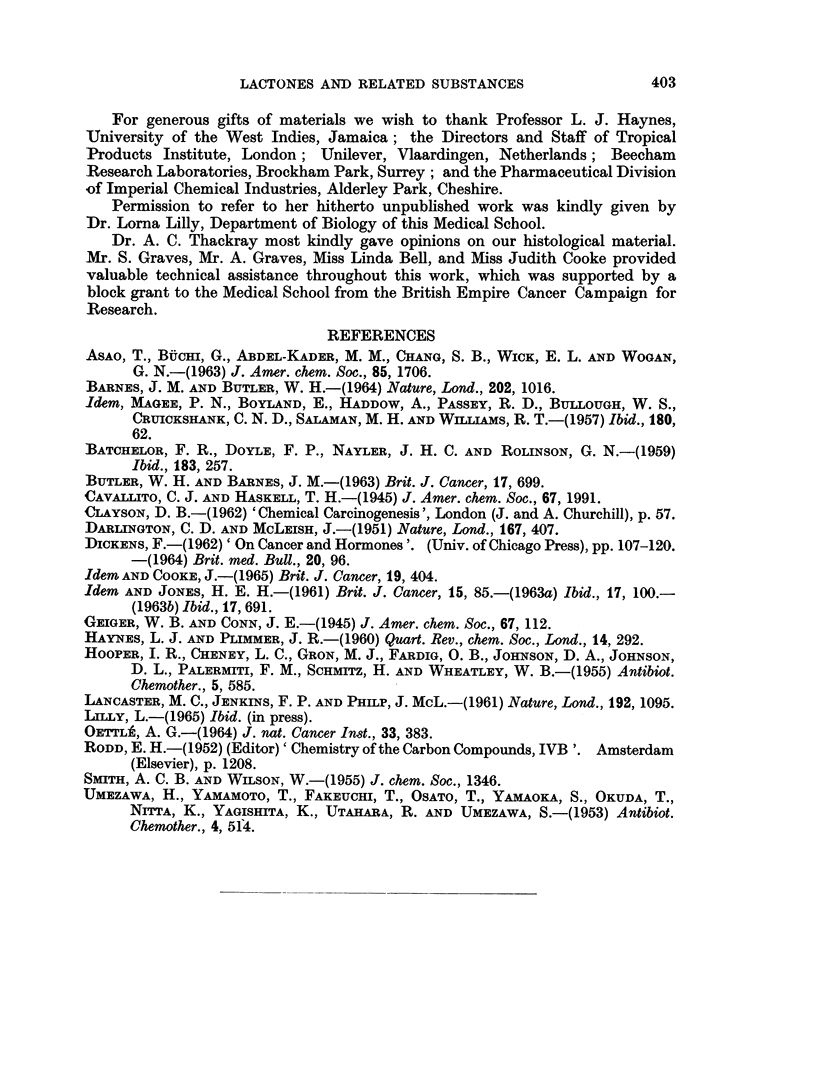# Further Studies on the Carcinogenic Action of Certain Lactones and Related Substances in the Rat and Mouse

**DOI:** 10.1038/bjc.1965.47

**Published:** 1965-06

**Authors:** F. Dickens, H. E. H. Jones


### FURTHER STUDIES ON THE CARCINOGENIC ACTION OF CERTAIN LACTONES AND RELATED SUBSTANCES IN THE RAT AND MOUSE

F. DICKENS AND H. E. H. JONES

*From the Courtauld Institute of Biochemistry, Middlesex Hospital Medical School, London, W.1*

Received for publication January 12, 1965

IN previous publications (Dickens and Jones, 1961, 1963*a*, 1963*b*; Dickens, 1962, 1964) we have described the carcinogenic action after repeated subcutaneous injection in the rat of a number of chemically reactive lactones and related compounds. Certain chemical features were prominent among the actively carcinogenic members of this series, including: (a) the presence of a four membered heterocyclic ring or (b) the presence of an $\alpha\beta$-unsaturated bond in a 5 or 6-membered lactone ring, especially when associated with an extracyclic double bond at the 4-position, (c) a cyclic anhydride of the type of maleic anhydride, which has $\alpha\beta$-unsaturation conjugated with the presence of the two carbonyl groups in the anhydride ring.

On the other hand, in the lactones hitherto tested, absence of $\alpha\beta$-unsaturation, as in the $\beta\gamma$-unsaturated $\alpha$-angelica lactone and in the fully saturated $\gamma$-butyro lactone, or opening of the lactone ring by hydrolysis, resulted in loss of carcinogenicity.

In the case of the remarkably highly carcinogenic unsaturated lactone represented by the mould-product aflatoxin, the chemical structure of this compound was not known at the time when groundnut meal infected with *Aspergillus flavus* was first shown to constitute a hepato-carcinogenic diet for the rat (Lancaster, Jenkins and Philp, 1961; see also Butler and Barnes, 1963). The purified toxin (mixed aflatoxins $B_1$ and $G_1$), made available to us by the Tropical Products Institute, D.S.I.R., London, was shown to produce local malignant tumours at the injection site in rats in doses as low as 50 $\mu$g (Dickens and Jones, 1963*b*). Later, feeding similar purified toxin to rats was shown to lead to hepatoma formation, like the contaminated meal (Barnes and Butler, 1964). Chemical studies at the Massachusetts Institute of Technology have now revealed that both purified aflatoxins $B_1$ and $G_1$ contain respectively one or two 6-membered lactone rings possessing $\alpha\beta$-unsaturation which is conjugated directly with further double bonds in the molecule (Asao *et al.*, 1963; see also Dickens and Jones, 1963*b*). Whether the remaining parts of these fairly complex molecules are involved in their carcinogenicity remains to be studied.

In the present paper we have extended the above observations in various directions. In the first place, we have added further tests on coumarin (I) (since the coumarin ring is contained in the aflatoxins), and on coumalic acid (II) a near relative: unfortunately both compounds proved highly toxic. The same applied to *N*-ethyl maleimide (III), a nitrogen analogue of maleic anhydride and a powerful sulphydryl-reacting material.

I

II

III

IV

V

VI

VII

VIII

IX

X

XI

XII

XIII

XIV

XV

We also included the substance maleic hydrazide (IV) which can be regarded as maleic anhydride with the ring-oxygen replaced by the hydrazide (—NH—NH—) group. $\alpha\beta$-Dimethyl maleic anhydride (V) is of interest because it is quite stable in presence of water, unlike maleic anhydride which rapidly hydrolyses. The substance bovolide (VI) is closely related chemically to $\alpha\beta$-dimethyl maleic anhydride (V) being an $\alpha\beta$–$\gamma\delta$-unsaturated five-membered lactone with an alkyl side chain ($C_5H_{10}$) attached by means of a double bond at the $\gamma$-position : it is a naturally-occurring flavouring material present in milk-fat and butter. In relation to maleic anhydride, the fully saturated succinic anhydride (VII) was tested. $\beta$-Angelica lactone (VIII) and $\alpha$-methyl tetronic acid (IX) represent two further $\alpha\beta$-unsaturated 5-membered lactones, the latter being related to ascorbic acid, while the cancer-inhibitory antibiotic substance sarkomycin (sodium salt; X; Umezawa *et al.*, 1953) has an interesting structure with the cyclopentanone carbonyl group conjugated with an external methylene group at the $\alpha$-position. Vinylene carbonate (XI) is an inversion of the structure occurring in maleic anhydride, while phenyl vinyl ketone (XII) is a highly reactive conjugated ketone which like the chemically related penicillic acid (open-chain form, formula XIII) shows some bacteriostatic properties (Geiger and Conn, 1945; Cavallito and Haskell, 1945.)

Finally, 6-aminopenicillanic acid (XIV) was included because of our earlier findings (Dickens and Jones, 1961; 1963*a*) that the benzyl derivative of this compound—penicillin G (sodium benzyl penicillin)—produced sarcomas after its prolonged subcutaneous injection into rats.

In view of the potential importance of aflatoxin in the remarkably high incidence of primary liver cancer in certain moist tropical countries, where the mould *Aspergillus flavus* probably flourishes (Oettlé, 1964), we have now studied; (a) the relative carcinogenic doses of mixed aflatoxins $B_1$ and $G_1$ given subcutaneously to rats and mice, and (b) the carcinogenicity of the isolated pure components, aflatoxin $B_1$ and aflatoxin $G_1$, in the rat. In experiments now in progress, these results are being compared with the oral toxicity, by administration in the animals' drinking water.

Since the statement has been repeatedly made that the subcutaneous tissues of the rat are unduly susceptible to tumour induction after subcutaneous injections, we have repeated, on the mouse, several of our earlier experiments on cancer induction in rats by various lactones. Our results give no support to this widespread but, in our view, unfounded belief.

### EXPERIMENTAL

### *Materials*

The following gifts are gratefully acknowledged.

Aflatoxins $B_1$ and $G_1$ (mixed sample, containing respectively 38 and 56 per cent of these components, as described by Dickens and Jones, 1963*b*), and later sufficient pure aflatoxin $B_1$ and aflatoxin $G_1$ for separate testing, were generously provided by Dr. B. F. Nesbitt, Tropical Products Institute, Department of Scientific and Industrial Research, London. (The formulae of these materials are given in Dickens and Jones, 1963*b*).

Bovolide (VI) and $\alpha\beta$-dimethyl maleic anhydride (V) were given by Dr. J. G. Keppler, Unilever Research Laboratorium, Vlaardingen, Rotterdam. Bovolide was provided as a 25 per cent solution in arachis (groundnut) oil; this stock was kept at +4° C. and was diluted 62 times with arachis oil B.P. before injecting 0·5 ml. (containing 2 mg. of VI) into rats. The substance occurs naturally in cow's butter to the extent of 0·1 to 0·5 parts per million, and is one of the substances responsible for the pleasing aroma of fresh butter.

$\alpha$-Methyltetronic acid (IX) was a gift from Professor L. J. Haynes, University of the West Indies, Jamaica (see Haynes and Plimmer, 1960). 6-Aminopenicillanic acid (XIV) was provided by Dr. F. P. Doyle of Beecham Research Laboratories (see Batchelor, Doyle, Naylor and Rolinson, 1959).

Phenyl vinyl ketone (acrylophenone, XII) was prepared from $\beta$-dimethylaminopropiophenone hydrochloride kindly supplied by Dr. B. W. Langley, Imperial Chemical Industries, Pharmaceuticals Division. This was converted on prolonged steam-distillation into phenyl vinyl ketone and dimethylamine hydrochloride as described by Smith and Wilson (1955) and the ketone was immediately dissolved (2 g./50 ml.) in arachis oil. This stock solution was kept at 4° C. and diluted with oil before use.

Penicillic acid (XIII, the same sample as shown to be carcinogenic by Dickens and Jones, 1961) was a gift from Professor J. H. Birkinshaw, School of Hygiene and Tropical Medicine, London.

$\beta$-Angelica lactone (VIII, L. Light and Co.) required fractional distillation *in vacuo* for purification, the fraction boiling at 68–70° C./1 mm. Hg being that used. $\beta$-Propiolactone (XV, L. Light and Co.) was redistilled at 51° C./10 mm. Hg.

Sarkomycin (X, sodium salt) was purchased from L. Light and Co. Its chemical constitution is described by Hooper *et al.* (1955). It was insoluble in arachis oil in which it was therefore injected as a very fine suspension.

The remaining substances were purchased, as the purest commercially available grades, from L. Light and Co. or British Drug Houses Ltd. Maleic hydrazide (purity checked by melting point and by analysis for C, H and N by Drs. Weiler and Strauss, Oxford), was injected as a finely-ground suspension in arachis oil ; it was used as the free substance and not as a salt.

### *Animal experiments*

The details of injection and histological techniques have already been described (Dickens and Jones, 1961). Twice-weekly injections of all substances were made for up to 65 weeks as nearly as possible into the same site on the right flank of the animals. Groups of 6 rats, each weighing about 100 g. at the beginning of the experiment, or 20 mice, Tuck No. 1 strain weighing 20–25 g., were used. In all cases the injections were in 0·5 ml. arachis oil into the rats, and 0·1 ml. arachis oil into the mice. In most cases 2 mg. of the test substance was injected on each occasion into the rats. Groups of rats were also given 10 $\mu$g. and 2 $\mu$g. doses of mixed aflatoxins, or 20 $\mu$g. doses of the purified aflatoxins, $B_1$ and $G_1$. Groups of mice were dosed with 10 $\mu$g. mixed aflatoxins, 200 $\mu$g. penicillic acid or 20 $\mu$g. $\beta$-propiolactone in oil according to the same injection schedule.

Substances which were not completely soluble in the oil were injected as a fine suspension, and injections were temporarily discontinued in animals which showed ulceration at the injection site. Each tumour was examined histologically and usually by transplantation into young female animals, and surviving animals which did not develop tumours were autopsied when killed 106 weeks after the first injection.

### RESULTS

### *New compounds tested* (see Tables I and II)

No tumours were obtained in rats treated twice-weekly for 65 weeks with 2 mg. of *N*-ethyl maleimide, coumalic acid or coumarin. These substances are somewhat toxic to the animals ; the mortality was high and many injections of *N*-ethyl maleimide had to be withheld because of ulceration at the injection sites.

Injections of 2 mg. sarkomycin, 6-aminopenicillanic acid and $\beta$-angelica lactone each induced only one tumour at the site of injections. This suggests a low order

TABLE I.—*The Carcinogenic Action of Compounds Administered Twice Weekly by Subcutaneous Injection in Oil to Male Rats*

| Substance Tested* | Duration of treatment (weeks) | Earliest appearance of tumours (weeks) | Number of rats alive at time of tumour appearance or at end of experiment | Number of rats with local tumours | Other tumours found at autopsy | Total period observed (weeks) |
|---|---|---|---|---|---|---|
| Arachis oil-controls | 45 | — | 3 | 0 | 0 | 45 |
| | 65 | — | 3 | 0 | 0 | 106 |
| (Previous results Dickens and Jones, 1963*a*) | 54–61 | — | 18 | 0 | 1 thyroid : 1 secondary thyroid in adrenal : 1 in thorax | 54–107 |
| Coumarin | 65 | — | 1 (toxic) | 0 | 0 | 108 |
| Coumalic acid | 65 | — | 2 (toxic) | 0 | 0 | 106 |
| Sarkomycin | 65 | 42 | 6 | 1 | 0 | 106 |
| 6-Aminopenicillanic acid | 65 | 84 | 6 | 1 | 0 | 106 |
| β-Angelica lactone | 65 | 104 | 1 (toxic) | 1 | 0 | 104 |
| Succinic anhydride | 65 | 93 | 3 | 3 | 0 | 106 |
| αβ-Dimethyl maleic anhydride | 65 | 76 | 5 | 3 | 0 | 104 |
| Maleic hydrazide | 65 | 84 | 6 | 3 | 1 hepatoma | 106 |
| Bovolide | 65 | 87 | 5 | 5 | 0 | 106 |
| *N*-ethyl maleimide | 65 | — | 1 (toxic) | 0 | 0 | 106 |
| Phenyl vinyl ketone | 65 | 63 | 4 | 3 | 0 | 106 |
| Vinylene carbonate | 65 | 44 | 6 | 6 | 0 | 84 |
| α-Methyl tetronic acid | 65 | 63 | 4 | 2 | 0 | 99 |

* All animals received 0·5 ml. arachis oil ± 2 mg. stated substance at each injection.

of carcinogenic potency, particularly for 6-aminopenicillanic acid which gave rise to a tumour, histologically a sarcoma, though it failed to grow in any of the rats to which it was transplanted.

The other compounds tested gave rise to a significant number of tumours in the injected rats. α-Methyl tetronic acid induced 2 sarcomas in 4 animals which survived more than 63 weeks, though these were only feebly malignant by the criteria of histology and transplantation. Vinylene carbonate gave rise to local tumours in all the injected rats with a latent period of only 44 weeks ; many of these were capable of active growth when transplanted into other rats. Three sarcomas, all capable of successful transplantation, arose in rats given repeated injections of 2 mg. maleic hydrazide in oil for 65 weeks. These tumours were first seen 84, 95 and 104 weeks after the first injection and were histologically described as proliferating sarcomas or fibrosarcomas. The last rat to develop a tumour locally had in addition, a 1·5 cm. diameter nodule of hepatoma, free of cholangioma, in the liver. Succinic anhydride induced local transplantable tumours in all rats which survived more than 93 weeks. All rats treated with 2 mg. of bovolide repeatedly also developed transplantable sarcomas at the site of the injections after 87 weeks. A derivative of maleic anhydride, αβ-dimethyl maleic anhydride, induced the development of sarcomas at the injection site in 3 out of 5 rats which survived for more than 76 weeks after the first injection. Two of these tumours grew after transplantation. Phenyl vinyl ketone first induced a sarcoma in

TABLE II.—*Tumour Characteristics and Time of Appearance in Rats Injected with Lactones and Related Substances*

| Substance injected | Total dose (mg.) | Development time (weeks) | Weight of tumour (g.) | Histology of tumour | Transplants (takes/No. of rats) |
|---|---|---|---|---|---|
| Sarkomycin | 168 | 42 | 58 | Myxosarcoma | 1/5 |
| Phenyl vinyl ketone | 252 | 63 | 43 | Fibrosarcoma | 2/6 |
| | 260 | 106 | ? | Sarcoma | N/A |
| | 260 | 106 | 37 | Fibrosarcoma | 1/6 |
| Succinic anhydride | 260 | 93 | 20 | Sarcoma | 1/5 |
| | 260 | 104 | 48 | Fibrosarcoma | 1/6 |
| | 260 | 106 | 1 | Fibrosarcoma | N/A |
| Bovolide | 260 | 87 | ? | Fibrosarcoma | 2/6 |
| | 260 | 97 | 57 | Fibrosarcoma | 1/6 |
| | 260 | 97 | 17 | Fibrosarcoma | 0/6 |
| | 260 | 101 | 56 | Fibrosarcoma | 3/6 |
| | 260 | 104 | 28 | Fibrosarcoma | 0/6 |
| αβ-Dimethyl maleic anhydride | 260 | 76 | 140 | Fibrosarcoma | 2/6 |
| | 260 | 95 | 22 | Sarcoma | 0/6 |
| | 260 | 104 | 40 | Spindle cell sarcoma | 1/6 |
| Maleic hydrazide | 260 | 84 | 35 | Sarcoma | 5/6 |
| | 260 | 95 | 29 | Fibrosarcoma | 1/6 |
| | 260 | 104 | 20 | Fibrosarcoma | 2/6 |
| | | | | Hepatoma | N/A |
| 6-Amino penicillanic acid | 260 | 84 | 16 | Sarcoma | 0/6 |
| Vinylene carbonate | 176 | 44 | 19 | Fibrosarcoma | 1/6 |
| | 200 | 50 | 41 | Sarcoma | 0/6 |
| | 216 | 54 | 30 | Fibrosarcoma | 0/6 |
| | 224 | 56 | 12 | Fibrosarcoma | 2/6 |
| | 224 | 56 | 100 | Sarcoma | 4/4 |
| | 260 | 77 | 23 | Fibrosarcoma | 4/6 |
| α-Methyl tetronic acid | 260 | 74 | 30 | Sarcoma | 0/6 |
| | 260 | 95 | 11 | Fibrosarcoma | 0/6 |
| β-Angelica lactone | 260 | 104 | 43 | Sarcoma | 2/6 |

* N/A = not attempted treated rats 63 weeks after the first injection and 3 of 4 animals alive at that time eventually bore transplantable tumours.

*Aflatoxin-treated rats and mice* (Table III)

The results obtained in rats given subcutaneous injections of 500 μg. of mixed aflatoxins twice weekly for only 8 weeks, and 50 μg. twice weekly throughout the experiment have already been described (Dickens and Jones, 1963*b*). Later experiments show that as little as 2 μg. of the mixed aflatoxins injected repeatedly gives rise to tumours in the rats, but with a significantly longer latent period than larger amounts of the material. Doses of 10 μg. were as effective as 50 μg. doses and gave tumours in all the animals with an almost identical latent period of 24 weeks.

Aflatoxin $B_1$ was able to induce tumours in more rats and more quickly than equal amounts (20 μg. doses) of aflatoxin $G_1$.

Most of the mice injected repeatedly with 10 μg. of the mixed aflatoxins developed sarcomas, the first tumour appearing 23 weeks from the time of the

TABLE III.—*Studies on the Carcinogenicity of Aflatoxins Administered Twice Weekly by Subcutaneous Injection in Oil to Rats and Mice*

| Species | Aflatoxin injected | Amount at each injection (μg.) | Earliest appearance of tumours (weeks) | Number of animals at time of tumour appearance | Number of animals developing local tumours | Other tumours found at autopsy | Time of appearance of last tumour (weeks) |
|---|---|---|---|---|---|---|---|
| Rats | Mixed $B_1$ and $G_1$ | 500 (for 8 weeks only) | 20 | 5 | 5 | 0 | 35 |
| | Mixed $B_1$ and $G_1$ | 50 | 21 | 6 | 6 | 0 | 60 |
| | Mixed $B_1$ and $G_1$ | 10 | 24 | 6 | 6 | 0 | 41 |
| | Mixed $B_1$ and $G_1$ | 2 | 44 | 6 | 5 | 0 | 69 (1 rat still alive) |
| | $B_1$ | 20 | 18 | 6 | 6 | 0 | 37 |
| | $G_1$ | 20 | 30 | 6 | 4 | 0 | 50 (1 rat still alive) |
| Mice | Mixed $B_1$ and $G_1$ | 10 | 23 | 17 | 15 | 0 | 76 |

first injection. This result is very similar to that obtained with 10 μg. doses of the same material in rats (Table III).

*Comparative action of some carcinogenic lactones in rats and mice*

The incidence of tumours observed in rats after repeated injections of arachis oil, β-propiolactone (100 μg.) in oil, penicillic acid (1 mg.) in oil and aflatoxins $B_1$ and $G_1$ in oil has been reported (Dickens and Jones 1961, 1963*a*, 1963*b*, and this paper).

In mice, one tumour which was histologically a non-secretory mammary adenoma has appeared at the injection site after 69 weeks treatment with arachis oil alone. The occurrence in rats of local tumours after the injection of arachis oil alone has never been observed by us (Table I). All three chemical compounds found to be carcinogenic in the rat were also effective in the mouse (Table IV).

TABLE IV.—*Comparison of Carcinogenic Action of some Lactones and Related Substances Administered by Subcutaneous Injection Twice Weekly to Rats and Mice*

The amount of each injection was contained in arachis oil, 0·5 ml. for rats, 0·1 ml. for mice

| Experimental animals | Substance injected | Amount at each injection | Number of survivors at time of first tumour appearance or at end of experiment | Number of animals developing local tumours | Earliest appearance of tumours (weeks) | Total period observed (weeks) |
|---|---|---|---|---|---|---|
| Rats | Arachis oil | 0·5 ml. | 24 | 0 | — | 45–106 |
| Mice | Arachis oil | 0·1 ml. | 19 | 1* | 69 | 72 |
| Rats | β-Propiolactone | 100 μg. | 4 | 4 | 25 | 34 |
| Mice | β-Propiolactone | 20 μg. | 20 | 10 | 43 | 81 |
| Rats | Penicillic acid | 1000 μg. | 4 | 4 | 48 | 67 |
| Mice | Penicillic acid | 200 μg. | 19 | 6 | 38 | 81 |
| Rats | Aflatoxins ($B_1$ and $G_1$) | 10 μg. | 6 | 6 | 24 | 41 |
| Mice | Aflatoxins ($B_1$ and $G_1$) | 10 μg. | 17 | 15 | 23 | 76 |

* A mammary adenoma : see text.

The doses chosen for $\beta$-propiolactone and penicillic acid were smaller for mice than rats, being approximately proportional to their initial body weight. Under these conditions, fewer tumours were obtained in the mice. But where, as with the aflatoxins (Table IV), equal doses were given to both species, the total carcinogenic response and the times of appearance of the tumours were closely similar.

### DISCUSSION

New compounds which have been shown to be actively carcinogenic in rats are phenyl vinyl ketone, succinic anhydride, maleic hydrazide, vinylene carbonate and $\alpha$-methyl tetronic acid. The appearance of one tumour in groups of rats treated with sarkomycin, 6-aminopenicillanic acid and $\beta$-angelica lactone would also suggest some carcinogenic activity by these compounds, but the small yield of tumours and the very long delay in their appearance, particularly with the latter two substances, indicate that such potential is very low. $\alpha$-Angelica lactone, possessing $\beta\gamma$-unsaturation, did not induce tumours on injection into rats in an experiment which lasted 100 weeks (Dickens and Jones, 1961), whilst purified $\beta$-angelica lactone tested in the present series gave only one tumour after 104 weeks. This latter compound possesses a double-bond in the $\alpha\beta$-position to the lactone carbonyl group and this is a type of structure which we have consistently found to be associated with carcinogenic activity (see Dickens, 1964). Owing to the extremely weak carcinogenic activity of $\beta$-angelica lactone, however, the correlation with chemical structure is not justifiable for these two compounds, though it is quite clearly shown by their next higher homologues (Dickens and Jones, 1961).

All the tumours which developed locally were histologically identified as sarcomas with variable amounts of collagen deposition, and some variability in cellular form. Many were considered to be malignant from histological criteria and the results in transplantation studies generally supported this. 6-Aminopenicillanic acid and $\alpha$-methyl tetronic acid, for instance, induced tumours which were not considered to be very malignant histologically and did not grow on transplantation, while the tumours obtained with bovolide, maleic hydrazide and vinylene carbonate were judged to be malignant on both grounds. The only tumour found at a site remote from the injections was borne by a rat which had been injected with maleic hydrazide and was killed because a sarcoma was growing at the injection site. The liver of this rat bore a hepatoma, and while it is not possible to say definitely whether it arose as a result of the treatment this seems highly probable since a large number of our rats more than 2 years old have been examined in the course of this series of experiments and spontaneous liver tumours have never been seen.

A carcinogenic property has not previously been demonstrated for maleic hydrazide and, in fact, Barnes *et al.* (1957) have shown that repeated once-weekly injections into rats of its diethanolamine salt or its sodium salt in water led to so few sarcomas at the injection site that in their view these substances could be considered to be harmless to animals. On the other hand it has been clearly shown that maleic hydrazide is capable of causing chromosome breakage in roots of *Vicia faba* (Darlington and McLeish, 1951), and this property is often associated with carcinogenicity. Under the conditions of our experiment the free substance, maleic hydrazide, injected in an oil medium twice-weekly into rats, definitely behaves as a carcinogen (Tables I and II). This result is of some importance in view of the widespread use of these compounds in agriculture, in order to suppress plant growth and the sprouting of potatoes. Our positive results with the pure maleic hydrazide may be considered to counteract to some extent the more reassuring negative experiments of Barnes *et al.* (1957), obtained with the salts of this compound, which appear to be the form in which this material is mainly used in agriculture. Since, however, these are salts of the acidic enolic form of maleic hydrazide (see Rodd, 1952), which in neutral aqueous solution would be formed immediately from maleic hydrazide, it would be surprising if the difference were merely due to the use of the sodium or ethanolamine salts in the experiments of Barnes *et al.* (1957). On the other hand their use of aqueous rather than oily solutions, and their practice of giving only one injection per week, may have resulted in such rapid elimination of the material from the injection site that the carcinogenic activity was not clearly demonstrable by them. Further work on all these points is clearly desirable.

The carcinogenicity of maleic anhydride has already been described (Dickens and Jones, 1963*a*) and in this study we have tested in addition to maleic hydrazide a number of substances which can be structurally compared with the anhydride. Succinic anhydride may of course be regarded as a hydrogenated maleic anhydride, whilst the substitution of the two hydrogen atoms by methyl groups gives rise to $\alpha\beta$-dimethyl maleic anhydride. Both of these derivatives, as well as maleic hydrazide, showed approximately the same carcinogenicity as maleic anhydride itself (compare Tables I and II, this paper, and Dickens and Jones, 1963*a*). This result with succinic anhydride shows that here the presence of $\alpha\beta$-unsaturation is not essential for carcinogenicity. However, it should be remembered that all anhydrides of this type are powerful acylating agents, and we have found (Dickens and Cooke, 1965) that both maleic and succinic anhydrides react vigorously with the sulphydryl group of cysteine in neutral aqueous solution, although only maleic anhydride gives an alkali-stable derivative, presumably by addition of cysteine to the double bond. We have also found that $\alpha\beta$-dimethyl maleic anhydride, in spite of being a carcinogen (Tables I and II), reacts only very slowly with the sulphydryl group of cysteine under similar conditions ; and this also applies to aflatoxin in our experience. These chemical findings are therefore difficult to correlate in any simple manner with carcinogenesis and some other possible mechanisms are discussed by Dickens (1964). Bovolide also can be fitted into this comparison since it is an $\alpha\beta$-dimethyl maleic anhydride derivative in which a five-carbon chain, bound by a double bond, replaces one of the oxygen atoms of a carbonyl group. This substance has also approximately the same carcinogenicity.

A further derivative in which the ring oxygen atom of maleic anhydride has been substituted by an *N*-ethyl group, *N*-ethyl maleimide, proved to be very toxic, but it did not induce tumours even in the one rat which survived to the end of the experiment, though this cannot be considered as significant.

The presence (Dickens and Jones, 1961, 1963*a*) of one or more double bonds conjugated with a carbonyl in the ring structure of the carcinogenic $\gamma$-lactones and in a $\delta$-lactone (parasorbic acid) led to the investigation of the effect of injecting phenyl vinyl ketone (XII), in which somewhat similar conjugation of the double bonds with the carbonyl group is present in a ketone. Moreover, under its alternative name of acrylophenone, this compound is one of several unsaturated ketones found by Geiger and Conn (1945) to share with the unsaturated lactones patulin and penicillic acid the possession of marked antibacterial activity. We have shown that, like penicillin G, both of these latter antibiotics are carcinogenically active (Dickens and Jones 1961, 1963*a*), and these compounds all react chemically with sulphydryl groups quite readily (Cavallito and Haskell, 1945 ; see Dickens, 1964) whereby their antibiotic activity is lost.

Phenyl vinyl ketone proved to be fairly actively carcinogenic (Table I) producing after a latent period of 63 weeks tumours which were both histologically and on transplantation judged to be malignant (Table II).

We have considered that, in view of the extremely high carcinogenic potency of the aflatoxins (see below) the substances coumarin and coumalic acid, having chemical structures which are closely related to a part of the aflatoxin molecule, might also prove to be carcinogenic. This was not so (Table I), although both series of tests were hindered by the toxicity of these materials.

Previous studies (Dickens and Jones 1963*b*) have shown that the twice-weekly subcutaneous injection of doses as low as 50 $\mu$g. of the mixed purified aflatoxins $B_1$ and $G_1$ into rats gave malignant tumours at the injection site in all the animals. The present work (Table III) shows that the same result is obtained with 10 $\mu$g. doses, and that even 2 $\mu$g. doses of aflatoxin are carcinogenic for the rat, the induction period increasing from 24 weeks with 10 $\mu$g. doses to 44 weeks with the lower amount. In mice, repeated 10 $\mu$g. doses have given almost as high an incidence of local malignant tumours (induction period 23 weeks, essentially the same as in the rat) as that observed by us in rats (Table III).

Tested separately in rats (for carcinogenicity) in 20 $\mu$g. doses, the two main components of the toxin produced by *Aspergillus flavus*, namely aflatoxins $B_1$ and $G_1$ showed a significant difference in activity (Table III). Whereas with the component $B_1$ a phenomenally short induction period of only 18 weeks was observed, this was extended to 30 weeks with aflatoxin $G_1$, and the subsequent tumours also appeared more rapidly with the $B_1$ component. It is interesting to compare this higher carcinogenicity of aflatoxin $B_1$ relative to $G_1$, with the similar relative order of toxicities of these two compounds for day old ducklings, which are reported to be : $LD_{50}$ for $B_1$, 28 $\mu$g. (or 550 $\mu$g./kg. body weight) ; for $G_1$, 90 $\mu$g. (or 1700 $\mu$g./kg.) (Asao *et al.*, 1963).

A high ability of the aflatoxins to induce chromosome breakage in the root-tips of *Vicia faba* has recently been observed in the Department of Biology, Middlesex Hospital Medical School, by Dr. Lorna Lilly (1965), to whom we are indebted for permission to mention this work, at present in course of publication.

The comparison of the activity of three compounds previously shown to be carcinogenic in the rat ($\beta$-propiolactone, penicillic acid and aflatoxin) with their carcinogenic action in the mouse, has shown that all three substances are carcinogens for the subcutaneous tissues of both species (Table IV). Allowing for the fact that different doses were used and for the fact that the mouse experiments are as yet incomplete, it would appear that with the same absolute dosage (i.e. one not based on body weight of the animals) fairly closely similar carcinogenic effects are to be expected in both rat and mouse. The relative carcinogenic response is certainly not directly related to the size of the animal, and the induction time varies with each of the three substances studied, being more rapid in the rat, for $\beta$-propiolactone, in the mouse for penicillic acid, and closely similar in both species for the aflatoxins (Table IV).

Such observations do not, in our view, lend any support to the idea that the subcutaneous tissues of the rat are so unduly susceptible to carcinogenic agents as to render the rat an unsuitable test-object. Such statements (e.g. Clayson, 1962) appear to be based on insufficient experimental evidence in which a *direct* comparison of the rat and mouse has been attempted under similar conditions.

As we have repeatedly emphasized (Dickens and Jones, 1961, 1963*a*, 1963*b*) we have never seen the induction of a local tumour at the injection site after repeated twice-weekly doses of 0·5 ml. arachis oil in the rat for very long periods (cf. Table IV). In the current series of experiments, we have injected 0·1 ml. doses of the same oil subcutaneously into mice, and in this case have observed the presence of one local tumour—a mammary adenoma—in 19 survivors after 69 weeks ; spontaneous mammary tumours are known to occur in this strain of mouse. Much more information on the relative susceptibility of various species and strains of animal is clearly necessary even to begin to discuss this problem in general terms.

### SUMMARY

1. Tests for carcinogenic activity in animals after repeated twice-weekly subcutaneous injection in arachis oil, have been continued upon further substances related chemically to those already studied by Dickens and Jones (1961 ; 1963*a* ; 1963*b*).

2. No tumours resulted in rats from N-ethyl maleimide, coumarin or coumalic acid, these substances being toxic in the 2 mg. doses used.

3. A single tumour at the injection site was induced in rats by sarkomycin (sodium salt), 6-aminopenicillanic acid and $\beta$-angelica lactone : these were histologically sarcomatous growths.

4. Local tumours in 2 rats occurred with $\alpha$-methyl tetronic acid, a relative of ascorbic acid. Vinylene carbonate gave sarcomas in all surviving rats, while maleic hydrazide gave sarcomas in 3 rats together with a hepatoma in one of these animals ; all these sarcomas grew readily on transplantation. The positive result with free maleic hydrazide contrasts with negative results obtained with aqueous solution of the salts of this substance previously reported by other workers.

5. Succinic anhydride produced transplantable local tumours in all survivors. The same applied to bovolide, a natural flavouring of cow's butter. The related compound, $\alpha\beta$-dimethyl maleic anhydride also induced sarcomas in 3 of 5 rats, and phenyl vinyl ketone gave a similar result.

6. Further tests (cf. Dickens and Jones, 1963*b*) of purified aflatoxins (mixture of $B_1$ and $G_1$ components) showed that 10 $\mu$g. in repeated doses quickly induced sarcomata in all the rats, while even doses of 2 $\mu$g. were also carcinogenic but with a longer induction period. Tests of the separate components, $B_1$ and $G_1$, in 20 $\mu$g. doses showed that both were carcinogenic to rats, the greater activity of the $B_1$ corresponding with its known higher toxicity in birds.

7. A beginning has been made on the comparison of rats and mice for use in these tests of carcinogenicity. With arachis oil alone in rats no local tumours have arisen with repeated doses of 0·5 ml. whereas in mice given 0·1 ml. doses no sarcoma but only one mammary adenoma has been found.

Given subcutaneously to mice, $\beta$-propiolactone and penicillic acid were actively carcinogenic but rather less so than when five times the doses used were given to rats. On the other hand, equal doses (10 $\mu$g.) of mixed aflatoxins given to both mice and rats were almost identically carcinogenically active in both species.

For generous gifts of materials we wish to thank Professor L. J. Haynes, University of the West Indies, Jamaica ; the Directors and Staff of Tropical Products Institute, London ; Unilever, Vlaardingen, Netherlands ; Beecham Research Laboratories, Brockham Park, Surrey ; and the Pharmaceutical Division of Imperial Chemical Industries, Alderley Park, Cheshire.

Permission to refer to her hitherto unpublished work was kindly given by Dr. Lorna Lilly, Department of Biology of this Medical School.

Dr. A. C. Thackray most kindly gave opinions on our histological material. Mr. S. Graves, Mr. A. Graves, Miss Linda Bell, and Miss Judith Cooke provided valuable technical assistance throughout this work, which was supported by a block grant to the Medical School from the British Empire Cancer Campaign for Research.

### REFERENCES

ASAO, T., BÜCHI, G., ABDEL-KADER, M. M., CHANG, S. B., WICK, E. L. AND WOGAN, G. N.—(1963) *J. Amer. chem. Soc.*, **85**, 1706.

BARNES, J. M. AND BUTLER, W. H.—(1964) *Nature, Lond.*, **202**, 1016.

*Idem*, MAGEE, P. N., BOYLAND, E., HADDOW, A., PASSEY, R. D., BULLOUGH, W. S., CRUICKSHANK, C. N. D., SALAMAN, M. H. AND WILLIAMS, R. T.—(1957) *Ibid.*, **180**, 62.

BATCHELOR, F. R., DOYLE, F. P., NAYLER, J. H. C. AND ROLINSON, G. N.—(1959) *Ibid.*, **183**, 257.

BUTLER, W. H. AND BARNES, J. M.—(1963) *Brit. J. Cancer*, **17**, 699.

CAVALLITO, C. J. AND HASKELL, T. H.—(1945) *J. Amer. chem. Soc.*, **67**, 1991.

CLAYSON, D. B.—(1962) 'Chemical Carcinogenesis', London (J. and A. Churchill), p. 57.

DARLINGTON, C. D. AND MCLEISH, J.—(1951) *Nature, Lond.*, **167**, 407.

DICKENS, F.—(1962) ' On Cancer and Hormones '. (Univ. of Chicago Press), pp. 107–120. —(1964) *Brit. med. Bull.*, **20**, 96.

*Idem* AND COOKE, J.—(1965) *Brit. J. Cancer*, **19**, 404.

*Idem* AND JONES, H. E. H.—(1961) *Brit. J. Cancer*, **15**, 85.—(1963*a*) *Ibid.*, **17**, 100.—(1963*b*) *Ibid.*, **17**, 691.

GEIGER, W. B. AND CONN, J. E.—(1945) *J. Amer. chem. Soc.*, **67**, 112.

HAYNES, L. J. AND PLIMMER, J. R.—(1960) *Quart. Rev., chem. Soc., Lond.*, **14**, 292.

HOOPER, I. R., CHENEY, L. C., GRON, M. J., FARDIG, O. B., JOHNSON, D. A., JOHNSON, D. L., PALERMITI, F. M., SCHMITZ, H. AND WHEATLEY, W. B.—(1955) *Antibiot. Chemother.*, **5**, 585.

LANCASTER, M. C., JENKINS, F. P. AND PHILP, J. MCL.—(1961) *Nature, Lond.*, **192**, 1095.

LILLY, L.—(1965) *Ibid.* (in press).

OETTLÉ, A. G.—(1964) *J. nat. Cancer Inst.*, **33**, 383.

RODD, E. H.—(1952) (Editor) ' Chemistry of the Carbon Compounds, IVB '. Amsterdam (Elsevier), p. 1208.

SMITH, A. C. B. AND WILSON, W.—(1955) *J. chem. Soc.*, 1346.

UMEZAWA, H., YAMAMOTO, T., FAKEUCHI, T., OSATO, T., YAMAOKA, S., OKUDA, T., NITTA, K., YAGISHITA, K., UTAHARA, R. AND UMEZAWA, S.—(1953) *Antibiot. Chemother.*, **4**, 514.